# In-peptide amino acid racemization via inter-residue oxazoline intermediates during acidic hydrolysis

**DOI:** 10.1007/s00726-021-02951-7

**Published:** 2021-02-13

**Authors:** Anders Broberg, Christina Nord, Jolanta J. Levenfors, Joakim Bjerketorp, Bengt Guss, Bo Öberg

**Affiliations:** 1 Department of Molecular Sciences, Uppsala BioCentrum, Swedish University of Agricultural Sciences, P.O. Box 7015, 750 07 Uppsala, Sweden; 2 Ultupharma AB, Södra Rudbecksgatan 13, 752 36 Uppsala, Sweden; 3 Department of Biomedical Sciences and Veterinary Public Health, Swedish University of Agricultural Sciences, P.O. Box 7036, 750 07 Uppsala, Sweden; 4 Department of Medicinal Chemistry, Uppsala University, P.O. Box 574, 751 23 Uppsala, Sweden

**Keywords:** Peptide hydrolysis, Racemization of amino acids, Oxazoline, 3-hydroxyvaline, *Pedobacter cryoconitis*

## Abstract

Isopedopeptins are antibiotic cyclic lipodepsipeptides containing the subsequence L-Thr—L-2,3-diaminopropanoic acid—D-Phe—L-Val/L-3-hydroxyvaline. Acidic hydrolysis of isopedopeptins in D_2_O showed the D-Phe residues to racemize extensively in peptides with *L-3-hydroxyvaline* but not in peptides with *L-Val*. Similarly, one Leu residue in pedopeptins, which are related peptides containing the subsequence Leu—2,3-diaminopropanoic acid—Leu—L-Val/L-3-hydroxyvaline, was found to racemize in peptides with *L-3-hydroxyvaline*. Model tetrapeptides, L-Ala—L-Phe—L-Val/3-hydroxyvaline—L-Ala, gave the corresponding results, i.e. racemization of L-Phe only when linked to a *L-3-hydroxyvaline*. We propose the racemization to proceed via an oxazoline intermediate involving Phe/Leu and the L-3-hydroxyvaline residues. The 3-hydroxyvaline residue may form a stable tertiary carbocation by loss of the sidechain hydroxyl group as water after protonation. Elimination of the Phe/Leu H-2 and ring-closure from the carbonyl oxygen onto the carbocation results in the suggested oxazoline intermediate. The reversed reaction leads to either retained or inversed configuration of Phe/Leu. Such racemization during acidic hydrolysis may occur whenever a 3-hydroxyvaline residue or any amino acid that can form a stable carbocation on the C-3, is present in a peptide. The proposed mechanism for racemization was supported by incorporation of ^18^O in the 3-hydroxyvaline sidechain when the acidic hydrolysis was performed in H_2_O/H_2_^18^O (1:1). The 2,3-diaminopropanoic residues of isopedopeptins and pedopeptins were also found to racemize during acidic hydrolysis, as previously described. Based on the results, the configuration of the Leu and 2,3-diaminopropanoic acid residues of the pedopeptins were reassigned to be L-Leu and D-Leu, and 2 × L-2,3-diaminopropanoic acid.

**Supplementary Information:**

The online version contains supplementary material available at 10.1007/s00726-021-02951-7.

## Introduction

Molecules produced by the metabolism of living organisms frequently contain one or more stereogenic carbon atom in their structures. Oligo- and polysaccharides are constructed from D- and/or L-monosaccharides and peptides may be constructed from L- and/or D-amino acids, and terpenes, polyketides, alkaloids or phenylpropanoids often contain several stereogenic carbon atoms. Thus, one crucial step in natural products characterization is the determination of the absolute configuration, and this is often the most challenging step of the structure elucidation. Consequently, many different experimental procedures have been developed for this purpose, including circular dichroism spectroscopy combined with e.g. the octant rule (Lightner 2000) or advanced quantum chemical calculations (Bringmann et al. 1997) and diastereomeric resolution based methods followed by NMR (Dale and Mosher 1973) or chromatographic analysis (Harada et al. 1995; Fujii et al. 1997). Chromatographic diastereomeric resolution-based methods are commonly applied on carbohydrates and peptides, and the first experimental step is to release the chiral monomeric building blocks from the parent compounds for subsequent derivatization and chromatographic analysis. Peptides are typically hydrolysed in strong mineral acids at elevated temperature for many hours, and for most standard amino acids, free or as building blocks of peptides or proteins, only a rather low frequency of racemization is observed in standard conditions for acidic hydrolysis. For example, Bayer et al. (1970) reported ca 1% racemization of Leu and < 0.5% racemization of Phe in a synthetic peptide as well as in bovine serum albumin (BSA) after treatment in 6 M HCl at 100 °C for 24 h (Bayer et al. 1970), whereas a more recent study showed ca 2% racemization of these amino acids when BSA was treated in 6 M HCl at 110 °C for 24 h (Kaiser and Benner 2005). There are, however, differences in racemization rates between different amino acids and between the same amino acid in different proteins (e.g. Kaiser and Benner 2005), and such differences have been attributed to inductive and steric effects, as well as intramolecular base action or solvation (Smith and De Sol 1980; Smith and Reddy 1989).

**Fig. 1:**
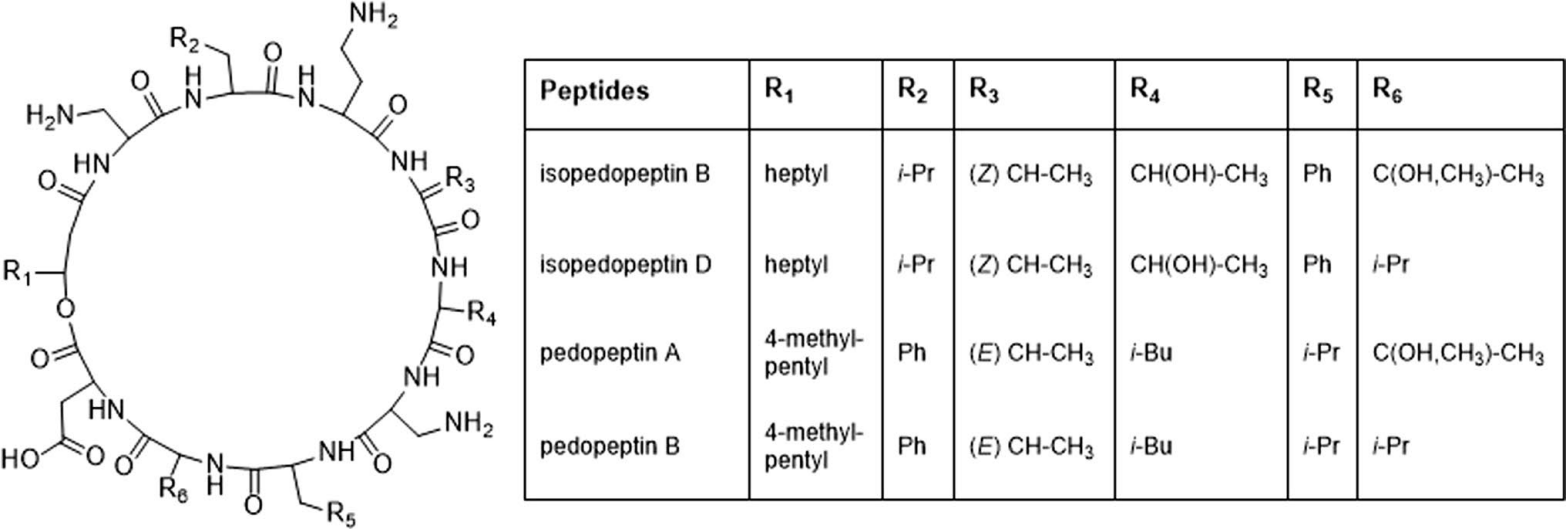
Structures of isopedopeptin B and D (Nord et al. 2020) and pedopeptin A and B (Hirota-Takahata et al. 2014) from *Pedobacter* sp

Isopedopeptin A-H are antibacterial cyclic lipodepsipeptides (Fig. 1) which are produced by the bacterium *Pedobacter cryoconitis* UP508 (Nord et al. 2020). These peptides have the general structure *cyclo*[(*R*)-3-hydroxyalkanoyl—L-2,3-diaminopropanoyl—L-Leu—L-2,4-diaminobutanoyl—(*Z*)-2-amino-2-butenoyl—L-Thr—L-2,3-diaminopropanoyl—D-Phe—L-3-hydroxyvaline/L-Val—L-Asp] (Fig. 1) (Nord et al. 2020). When characterizing these peptides, using the advanced Marfey’s method (Harada et al. 1995; Fujii et al. 1997), we noticed that L-2,3-diaminopropanoic acid (DAPA) racemized to a large extent during acidic hydrolysis of the peptides (Nord et al. 2020), in accord with previous findings (Kjær and Olesen Larsen 1959). However, we also noticed that the D-Phe residue of some of these peptides racemized to a substantial degree (Nord et al. 2020). In the present paper, we investigate the racemization of D-Phe in the isopedopeptins and the comparable racemization of D-Leu observed in the analogous pedopeptins (Fig. 1) (Hirota-Takahata et al. 2014), as well as racemization of L-Phe in synthetic model peptides. We present data supporting that the observed racemization occurs via inter-residue oxazoline intermediates, which may be formed in the presence of an amino acid, e.g. L-3-hydroxyvaline (OHVal) that can form a stable carbocation.

## Results and discussion

**Fig. 2:**
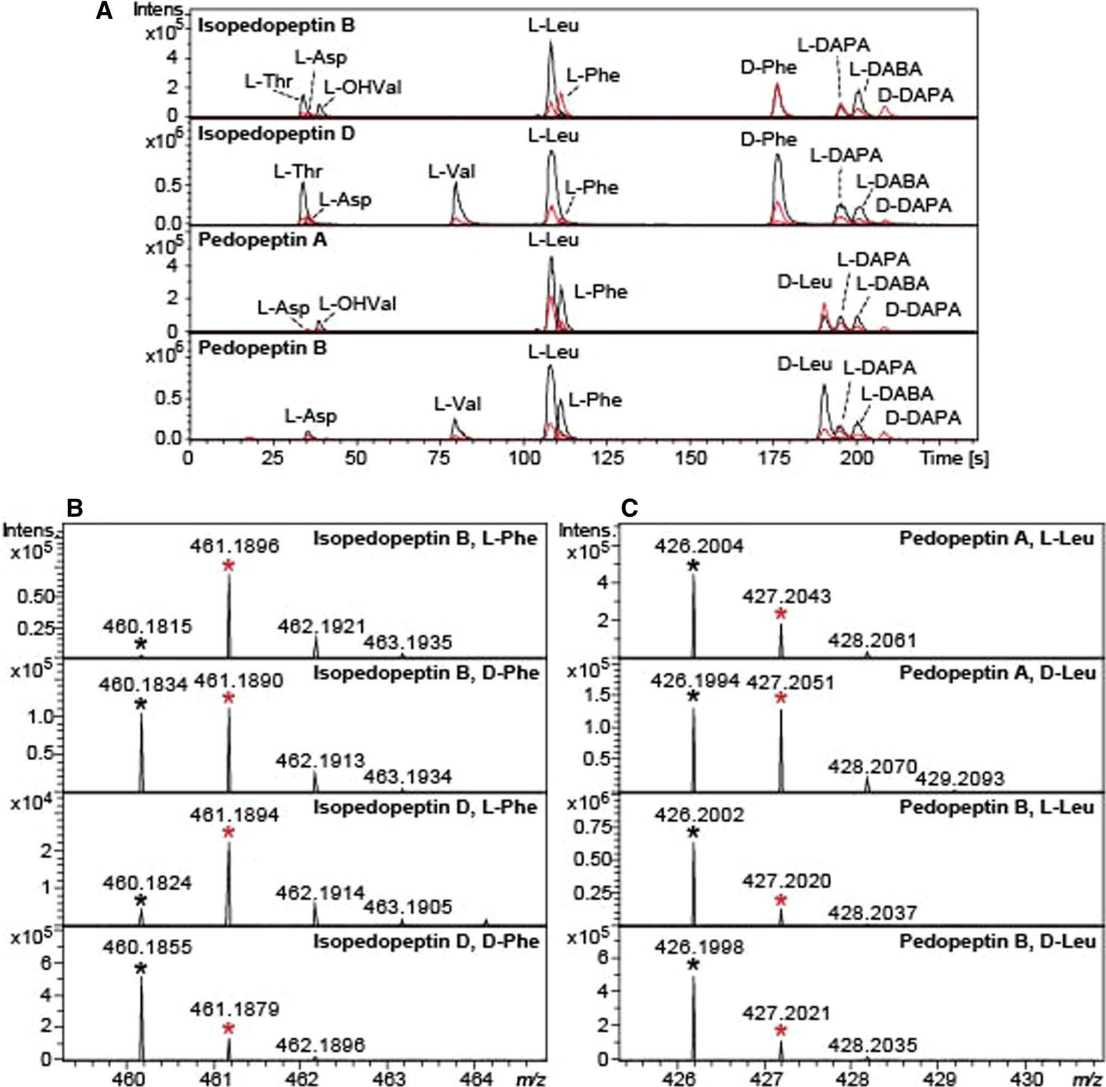
**a** Extracted-ion chromatograms from advanced Marfey’s analysis of isopedopeptin B and D, and pedopeptin A and B, with hydrolysis in 6 M DCl in D_2_O. Black chromatograms are extracted-ion chromatograms from the different monoisotopic ions of the FDLA derivatives and red chromatograms from the corresponding 1st isotope ions. **b** Selected regions of ESI-QTOF mass spectra for L- and D-Phe from isopedopeptin B and D. Monoisotopic ions are labelled with * and 1st isotope ions with  . **c** Selected regions of ESI-QTOF mass spectra for L- and D-Leu from pedopeptin A and B. Monoisotopic ions are labelled with * and 1st isotope ions with

When isopedopeptin B and D were subjected to acidic hydrolysis in D_2_O, and then analysed by the advanced Marfey’s method (Harada et al. 1995; Fujii et al. 1997), LCMS peaks from 1-fluoro-2,4-dinitrophenyl-5-L-leucinamide (L-FDLA) derivatives of L-Thr, L-Asp, L-OHVal, L-Leu, L- and D-Phe, L- and D-DAPA, as well as L-2,4-diaminobutanoic acid (L-DABA) were obtained (Fig. 2a). The rationale behind performing the acidic hydrolysis in D_2_O, was to detect racemization of amino acids, since racemization of α-amino acids will result in exchange of α-hydrogens to solvent-derived hydrogens. Thus, acidic hydrolysis in D_2_O will lead to the incorporation of one deuterium atom in racemized amino acids, which will result in increased intensity for the isotope ions, and in particular the 1st isotope ion, when analysed by MS.

When studying extracted ion-chromatograms for the monoisotopic ion and the 1st isotope ion for all amino acid derivatives and their mass spectra, it was evident that the 1st isotope ion of the L-Phe peak was much more intense than the monoisotopic ion, whereas in the peak of D-Phe, the two ions were of equal intensity (Fig. 2a and b). In comparison, for isopedopeptin D, which contains L-Val instead of L-OHVal, the peak from L-Phe was much smaller and the D-Phe peak was dominated by the monoisotopic ion (Fig. 2a and b). This indicates strongly that the D-Phe in isopedopeptin B undergoes substantial racemization during acidic hydrolysis, whereas the D-Phe in isopedopeptin D does not, and that the racemization includes hydrogen/deuterium exchange when the hydrolysis is done in D_2_O. Since the difference between these two peptides is a Phe neighbouring OHVal or Val, and the racemization was observed with a neighbouring OHVal, this amino acid residue is likely to be involved in a racemization process of Phe prior to hydrolysis of the peptide bond between these two amino acid residues. The related peptides pedopeptin A and B [*cyclo*(3-hydroxy-7-methyloctanoyl—DAPA—L-Phe—L-DABA—(*E*)-2-amino-2-butenoyl—Leu—DAPA—Leu—L-OHVal/Val—L-Asp)] (Fig. 1) (Hirota-Takahata et al. 2014), which differ by a OHVal or a Val residue, were analysed analogously. In these peptides, the OHVal and Val residues are neighbouring a Leu residue, and not a Phe residue, and thus, if the OHVal is involved in racemization of the Phe residue in isopedopeptin B, the OHVal should cause racemization of the neighbouring Leu residue of pedopeptin A, whereas this Leu should be unaffected in pedopeptin B. Indeed, the pedopeptin B analysis resulted in peaks from both L- and D-Leu, with normal ratios between the monoisotopic ions and the 1st isotope ions (Fig. 2a and c), suggesting the pedopeptins to contain both L- and D-Leu. On the contrary, pedopeptin A gave rise to a peak from D-Leu with similar intensity for the 1st isotope ion as for the monoisotopic ion, and a peak from L-Leu, with substantially larger 1st isotope ion compared to L-Leu from pedopeptin B (Fig. 2a and c). This is in line with the presence of both L- and D-Leu in pedopeptin A, and with racemization of the D-Leu due to a neighbouring OHVal residue.

**Fig. 3:**
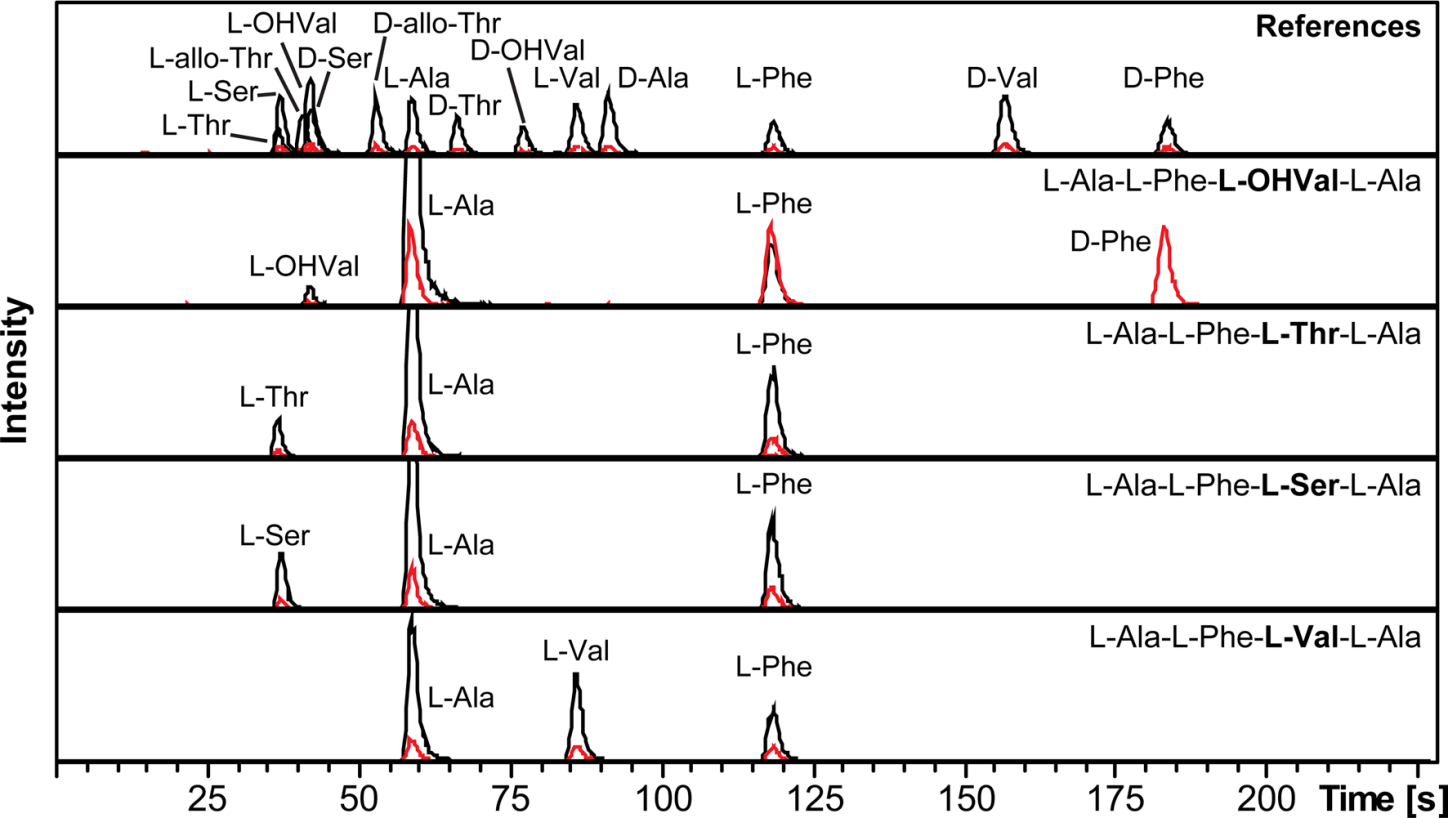
Extracted-ion chromatograms from advanced Marfey’s analysis of four model peptides, with hydrolysis in 6 M DCl in D_2_O. A reference mixture is shown on top and below are the results from analysis of the different peptides, with the variable amino acid indicated in bold typeface. Black chromatograms are extracted-ion chromatograms from the different monoisotopic ions of the FDLA derivatives and red chromatograms from the corresponding 1st isotope ions

Thus, the observed racemization of Phe and Leu in isopedopeptin B and pedopeptin A, respectively, appears to be associated with the presence of a neighbouring OHVal residue and not a Val residue on the carboxyl side of Phe or Leu, respectively. Two synthetic model peptides, L-Ala—L-Phe—*L-Val/OHVal*—L-Ala, were chosen to study this racemization further, and when subjected to the same treatment as the isopedopeptins and the pedopeptins, it became evident that the L-Phe in the peptide with L-OHVal was to a large extent racemized to D-Phe (Fig. 3). It was also noted that the D-Phe formed was dominated by the 1st isotope ion and that the L-Phe had equal contributions from the monoisotopic ion and the 1st isotope ion (Fig. 3). On the contrary, L-Phe of the peptide with L-Val, did not racemize at all, and the L-Phe was dominated by the monoisotopic ion.

**Fig. 4:**
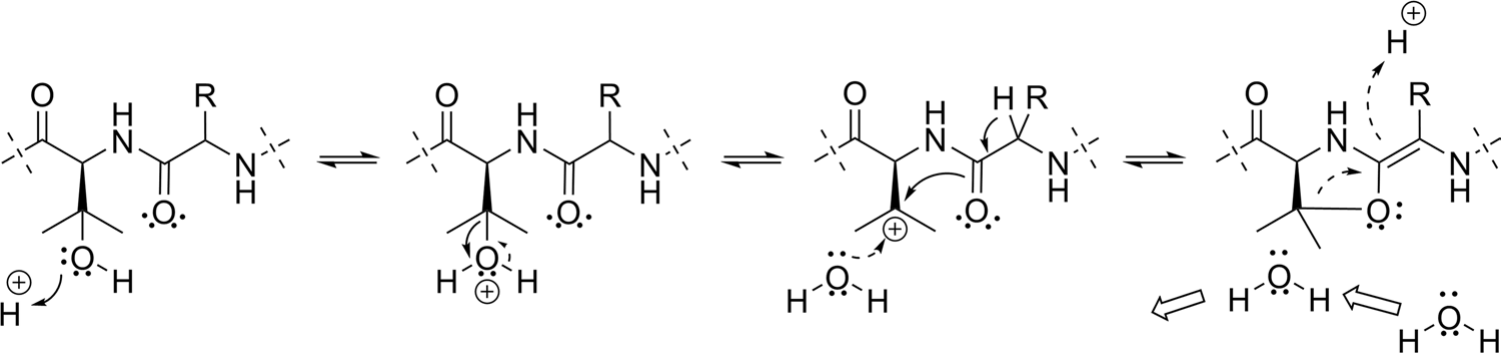
Proposed formation of in-peptide oxazoline intermediates involving 3-hydroxyvaline and Phe/Leu residues in isopedopeptin B (*R* = Bn) and pedopeptin A (*R* = *i*-Bu), respectively. Solid curved arrows explain forward reactions, and dashed curved arrows explain backward reactions. The half-life of the oxazoline determines if the 3-hydroxyvaline derived water molecule will be replaced by a solvent derived molecule via diffusion, before the 3-hydroxyvaline is reformed

The presence of the sidechain hydroxyl function in OHVal allows a relatively stable carbocation to be formed after protonation and loss of water (Fig. 4), which obviously cannot be formed from a Val residue. Potentially, such a carbocation may enable the formation of an oxazoline ring between the former OHVal residue and the neighbouring amino acid residue, i.e. Phe or Leu in this study, by loss of the Phe/Leu H-2 and subsequent ring-formation from the Phe/Leu carbonyl oxygen to the tertiary carbocation (Fig. 4). The reversed reaction, i.e. concomitant protonation from either side of the C-1/C-2 double bond of the former Phe/Leu residue and oxazoline ring opening, leads to either L- or D-Phe/Leu residues along with restoration of the tertiary carbocation, which then may reform the OHVal residue by addition of water (Fig. 4). To test if also L-Thr and L-Ser residues, i.e. residues with hydroxyl functions on C-3, may cause measurable racemization of a neighbouring L-Phe residue, the peptides L-Ala—L-Phe—*L-Thr/Ser*—L-Ala were subjected to the same treatment. However, in these experiments, no racemization of the L-Phe residues was detected, and the L-Phe isotope ratios were normal. The loss of water from Ser and Thr would result in primary and secondary carbocations, respectively, which are considerably less stable than a tertiary carbocation, and the formation of such ions are thus highly unlikely processes. However, it may still be possible for Ser and Thr residues to form oxazoline rings with the carbonyl oxygen of the Phe residue, by protonation of their hydroxyl groups followed by elimination of the Phe H-2 accompanied by nucleophilic displacement of the protonated hydroxyl functions by the carbonyl oxygen. In the present experiments, however, this alternative process does not appear to occur to a measureable extent.

**Fig. 5:**
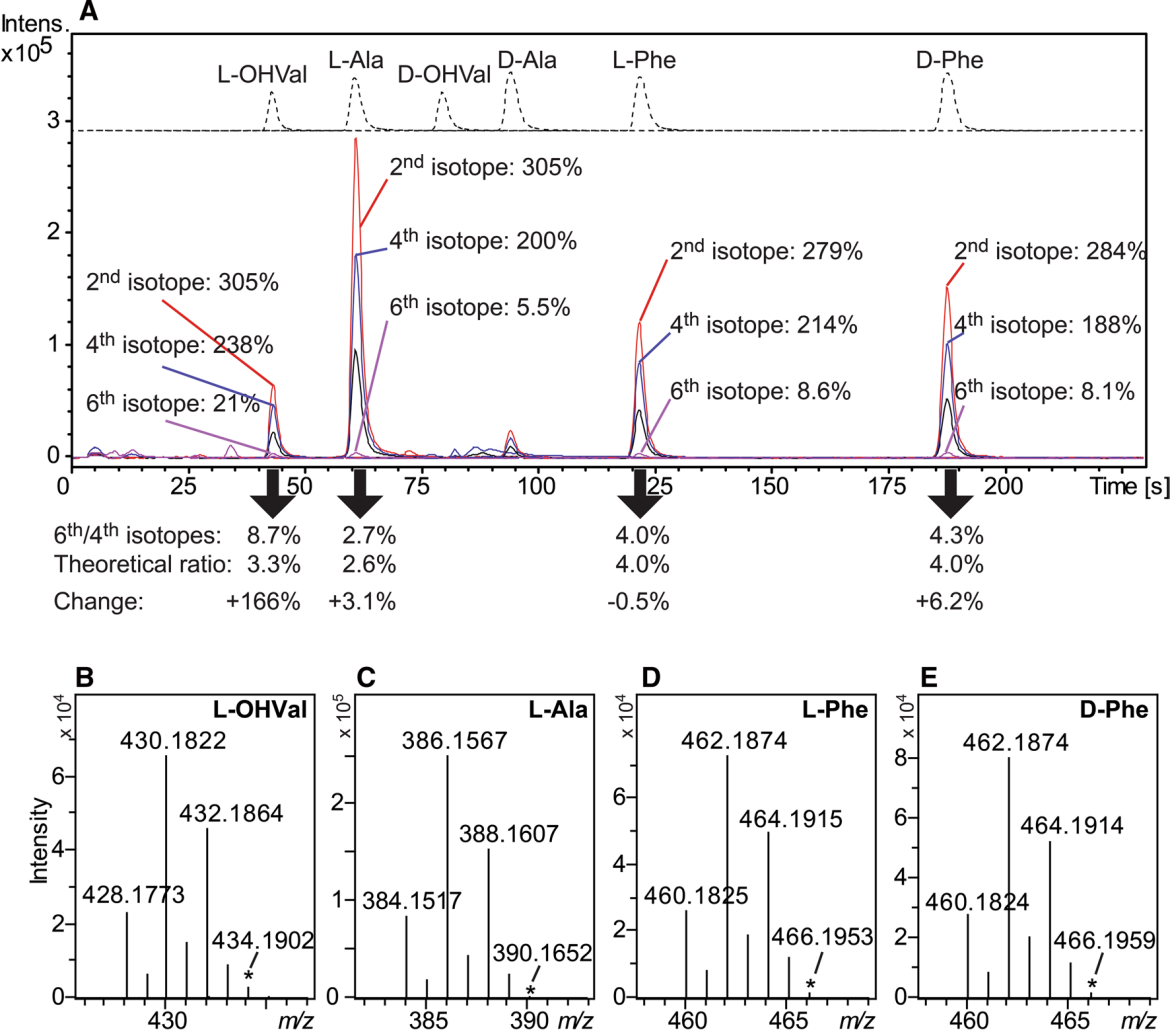
Advanced Marfey’s analysis of the peptide L-Ala – L-Phe – L-OHVal – L-Ala after hydrolysis in 6 M HCl in H_2_O/H_2_^18^O. **a** The extracted-ion chromatograms for L-FDLA derivatives of the released amino acids (black: monoisotopic ions; red: 2nd isotope ions; blue: 4th isotope ions; violet: 6th isotope ions) are shown on bottom, and reference amino acids are shown on top. The ratios between all displayed isotope ion-chromatograms peak areas and the respective monoisotopic ion-chromatogram peak areas are shown next to chromatographic peaks. Below the chromatographic peaks, the observed 6th/4th isotope ratios are shown, along with theoretical values and changes. **b**–**e** Selected regions of ESI-QTOF mass spectra for L-FDLA derivatives of L-OHVal, L-Ala, L-Phe and D-Phe, respectively. Each mass spectrum shows the monoisotopic ion and the 2nd, 4th and 6th isotope ions for the M + H+ ion. The 6th isotope ion is labelled with * in each spectrum

To further study the mechanism behind the observed racemization, the model peptide L-Ala—L-Phe—L-OHVal—L-Ala was subjected to advanced Marfey’s analysis with acidic hydrolysis in H_2_O/H_2_^18^O (1:1, Fig. 5a–e). During hydrolysis of the peptide bonds, H_2_^16^O or H_2_^18^O will be incorporated as a hydroxyl group into the carboxylic acid group of all released amino acids, and additionally, the incorporated carboxylic acid hydroxyl group may subsequently be exchanged by another water derived hydroxyl group. Furthermore, due to resonance and symmetry of the protonated carboxylic acid group [R–C = OH^+^(OH) ↔ R–C^+^(OH)_2_], the two oxygen atoms of each carboxylic acid group are identical, and thus, the isotopologues NH_2_-CHR-C^16^O_2_H, NH_2_-CHR-C^16^O^18^OH and NH_2_-CHR-C^18^O_2_H, may be formed during hydrolysis. For all amino acid L-FDLA derivatives, the [M + H]^+^ ion with the highest intensity was the 2nd isotope ion (279–305% of the monoisotopic ions, Fig. 5a) and the second most intense [M + H]^+^ ion was the 4th isotope ion (188–238% of the monoisotopic ions, Fig. 5a). Due to the similarity of these ratios for all amino acids, it is reasonable to assume that these ions are from the L-FDLA derivatives of the isotopologues NH_2_-CHR-C^16^O^18^OH and NH_2_-CHR-C^18^O_2_H, respectively, for all three amino acids, even if NH_2_-CH[C^18^OH(CH_3_,CH_3_)]-C^16^O_2_H and NH_2_-CH[C^18^OH(CH_3_,CH_3_)]-C^16^O^18^OH also are possible for L-OHVal. The incorporation of three ^18^O in the amino acids, in this case only possible for L-OHVal, would result in the isotopologue NH_2_-CH[C^18^OH(CH_3_,CH_3_)]-C^18^O_2_H which would be detected as increased intensity for the 6th isotope ion for the L-FDLA derivative. For the FDLA derivatives of L-OHVal, L-Ala, L-Phe and D-Phe, the intensities of the 6th isotope ion relative to the monoisotopic ion were 20.8%, 5.5%, 8.6% and 8.1%, respectively. As reasoned above, L-FDLA derivatives of the NH_2_-CHR-C^18^O_2_H isotopologues of OHVal, Ala and Phe will dominate the corresponding 4th isotope ions in the current analysis. If analysing pure ^18^O_2_-labelled OHVal, Ala, and Phe, as L-FDLA derivatives, the ratios 3.3%, 2.6% and 4.0%, respectively, would be expected between the 2nd isotope [M + H]^+^ ions (^13^C_2_^18^O_2_ ions) and the monoisotopic [M + H]^+^ ions (^18^O_2_ ions). In the current analysis, the ratios between the 6th and 4th isotope ions for the L-Ala, L-Phe and D-Phe FDLA derivatives, were within ± 6% of the above theoretical ratios. For L-OHVal, however, the observed ratio 6th/4th isotope ions showed a 166% increase compared to the theoretical value. This clearly suggests that NH_2_-CH[C^18^OH(CH_3_,CH_3_)]-C^18^O_2_H has been formed to some extent, which indicates that the above suggested L-OHVal-derived tertiary carbocation is present during the peptide hydrolysis.

The extent of ^18^O-incorporation in the OHVal residue sidechain, via the proposed tertiary carbocation, will partly depend on the half-life of the proposed oxazoline intermediate. If the oxazoline intermediate is long-lived, the water molecule eliminated from the OHVal residue will have time to diffuse away from the oxazoline and will be replaced by another water molecule from the solution, ^18^O-labelled or not (Fig. 4). In this scenario, racemization of Phe would often result in the incorporation of ^18^O in the OHVal sidechain, which would be observed as a high intensity 6th isotope ion. If short-lived, the eliminated water molecule will stay in close contact with the oxazoline, and will often be re-connected to C-3 to reform OHVal, and will rarely be replaced by a water molecule from the solution. In the current experiment, the peaks for L- and D-Phe were of similar intensity reflecting extensive Phe racemization in these conditions (Fig. 5a), but the 6th isotope ion for OHVal in the advanced Marfey’s analysis was only ca 20.8% of the monoisotopic ion. The rather low intensity of the 6th isotope ion for OHVal, compared to the monoisotopic ion, indicates that the oxazoline intermediates are rather short-lived and that the OHVal is mainly reformed without ^18^O-incorporation during racemization of the neighbouring Phe (Fig. 4).

The observation of this process in both natural peptides containing D-Phe or D-Leu linked to OHVal, such as the cyclic lipodepsipeptides isopedopeptin B (Nord et al. 2020) and pedopeptin A (Hirota-Takahata et al. 2014), as well as in a short linear synthetic model peptide with L-Phe linked to a OHVal residue, suggests that this process may occur in any peptide containing one or more OHVal residues, or other amino acid residues which may form a stable C-3 tertiary carbocation (e.g. 3-hydroxyisoleucine or similar). When screening the literature for peptides containing one or more OHVal structure elements neighbouring chiral α-amino acids, a number of peptides are found, including yakuamides (Ueoka et al. 2010), polytheonamides (Hamada et al. 2005), pedopeptins (Hirota-Takahata et al. 2014), and myxoprincomide (Cortina et al. 2012). However, in neither of these examples, problems with racemization of the neighbouring amino acids during determination of the absolute configuration were mentioned and discussed. The extent of the racemization is likely to depend on multiple factors, including the ease of hydrolysis of the peptide bond between the OHVal and its *N*-neighbour, since the racemization described here involves an oxazoline ring across this bond, and the overall peptide conformation, which must also allow a suitable orientation of the carbocation and the neighbouring carbonyl group for formation of the oxazoline ring.

When occurring, this racemization may obviously complicate the structure determination of peptides, even if the racemization can be traced using D_2_O as solvent for the hydrolysis, but it may also prove helpful. In the original description of the pedopeptin structures (Hirota-Takahata et al. 2014), only the absolute configuration of pedopeptin A was targeted by the advanced Marfey’s method, and pedopeptin A was described to contain both D- and L-Leu, but the location of the two residues was not determined. In our study, we detected substantial racemization of the D-Leu in pedopeptin A (OHVal containing) but not in pedopeptin B (Val containing), which strongly suggests that the D-Leu residue of pedopeptin A, and likely in all pedopeptins, is neighbouring the OHVal/Val residue, and the L-Leu is thus located between the DAPA and (*E*)-2-amino-2-butenoyl residues. In pedopeptin A, also the two DAPA residues were originally described to be present as both L- and D-isomers, but the positions of these residues were not determined. In our analyses, we likewise observed L- and D-isomers of DAPA from pedopeptin A and B, and from isopedopeptin B and D. The isotope patterns, however, strongly suggested that the D-DAPA residues were formed from L-DAPA residues by racemization, as highlighted by the presence of only the 1st isotope ion for the D-DAPA residues and mixtures of monoisotopic ions and 1st isotope ions for the L-DAPA residues (Fig. 2a). The racemization of DAPA in strong mineral acids has been observed previously (Kjær and Olesen Larsen 1959). Thus, we conclude that the pedopeptins contain two L-DAPA residues and no D-DAPA residue. Based on our observations, we propose the pedopeptin general structures to be *cyclo*[(*R*)-3-hydroxyalkanoyl—L-DAPA—L-Phe—L-DABA—(*E*)-2-amino-2-butenoyl—L-Leu—L-DAPA—D-Leu—L-Val/L-OHVal—L-Asp] (Fig. 1).

The amino acid L-Asp showed considerable incorporation of deuterium into the structure as illustrated by approximately equal intensities of the monoisotopic and 1st isotope ions (Fig. 2a). This did, however, not cause racemization of the amino acid, since no D-Asp was detected in the analysis. Instead, as previously described (Hill and Leach 1964), hydrogen/deuterium exchange takes places in the sidechain, in the CH_2_ group neighbouring the carboxylic acid group.

## Conclusion

We analysed natural and synthetic peptides containing D- or L-amino acids linked via their carboxyl groups to OHVal residues, and found that these D- or L- amino acids racemized extensively during acidic hydrolysis. In contrast, in analogous peptides with Val instead of OHVal, the corresponding racemization was not observed. We suggest this racemization to occur via oxazoline intermediates, formed by a mechanism involving an OHVal derived tertiary carbocation, which forms an oxazoline ring with the neighbouring amino acid upon elimination of H-2 of this amino acid. This racemization might occur during acidic hydrolysis of any peptide containing OHVal or other amino acids that may form a stable C-3 carbocation. Obviously, the racemization may complicate structure analysis of peptides, but in the case of the pedopeptins, our observation lead to the reassignment of the configuration of the Leu and DAPA residues in these peptides.

## Experimental

### General

Isopedopeptin B and D were obtained from previous work (Nord et al. 2020), whereas pedopeptin A and B, were obtained as described in the supplementary material file. The peptides L-Ala—L-Phe—L-Val/L-Ser/L-Thr—L-Ala were purchased from Genescript (Piscataway, NJ, USA), and the peptide L-Ala—L-Phe—L-OHVal—L-Ala was from Bachem (Bubendorf, BL, Switzerland). L-OHVal was from Acros Organics BVBA (Geel, Belgium), L-2,3-diaminopropanoic acid from Alfa Aesar (Kandel, Germany) and L-2,4-diaminobutanoic acid were from Sigma Aldrich (MO, USA). L- and DL-FDLA were synthesised as previously described (Marfey 1984), but with Leu-NH_2_ (Sigma Aldrich) instead of Ala-NH_2_. Concentrated DCl in D_2_O was from Cambridge Isotope Laboratories Inc (Tewksbury, MA, USA) and H_2_^18^O from Promochem Standard Supplies AB (Kungsbacka, Sweden). UHPLC-MS was performed on a maXis Impact Q-TOF MS (Bruker Daltonic GmbH, Bremen, Germany) connected to an Agilent 1290 Infinity II UHPLC (Agilent, Palo Alto, CA, USA). HPLC gradient grade MeCN (Sigma-Aldrich, St. Louis, MO, USA), deionized filtered water (Millipore, Billerica, MA, USA) and formic acid (Sigma Aldrich) were used for preparation of UHPLC solvents.

### Advanced Marfey’s analysis (Harada et al. 1995; Fujii et al. 1997)

Peptide samples (3 × 50 μg each for isopedopeptins, pedopeptins and for synthetic peptides) were hydrolysed in 100 μL 6 M DCl at 120 °C for 18 h, in evacuated glass ampoules. The synthetic peptide L-Ala—L-Phe—L-OHVal—Ala (4 × 50 μg) was also hydrolysed in 100 μL 6 M HCl in H_2_O/H_2_^18^O (1:1). The glass ampoules were opened, samples transferred to 1.5-mL plastic tubes and dried under N_2_. Each sample was then treated with 36 μL 1 M NaHCO_3_ and 90 μL L-FDLA (10 mg/mL acetone) for 1 h in 40 °C, and then 20 μL 2 M HCl (aq) was added. Following dilution with 500 μL MeOH and centrifugation (5 min, 13 000 rpm), the samples (2 μL injected) were analysed by UHPLC-MS on a reversed-phase column (2.1 × 50 mm, 1.5 μm, Accucore Vanquish, Thermo Scientific) using a gradient of MeCN in water, both with 0.2% formic acid (30–60% MeCN in 6 min at 0.9 mL/min). Reference samples were authentic L-amino acids derivatized with L-FDLA, whereas the chromatographic equivalents to DL mixtures were obtained by derivatizing L-amino acids with DL-FDLA. The MS was operated in positive mode with scanning of *m/z* 50−1500, and the mass spectra were calibrated against sodium formate clusters. Extracted-ion chromatograms corresponding the monoisotopic [M + H]^+^ ions and 1st isotope ions of the FDLA derivatives of relevant amino acids were constructed for the isopedopeptins, the pedopeptins, and for the synthetic peptides. For the sample hydrolysed in 6 M HCl in H_2_O/H_2_^18^O (1:1), the extracted-ion chromatograms for the monoisotopic [M + H]^+^ ions and the 2nd, 4th and 6th isotope ions were constructed and compared. Theoretical isotope ratios (2nd isotope ion/monoisotopic ion) for FDLA derivatives of ^18^O_2_-amino acids, were calculated with the software Compass IsotopePattern (Bruker Daltonic GmbH) using resolution 30,000.

## Supplementary Information

Below is the link to the electronic supplementary material.

Supplementary file1 (PDF 371 KB)

## Data Availability

Not applicable.
