# Supplementary material for: In-peptide amino acid racemization via inter-residue oxazoline intermediates during acidic hydrolysis
Source: Amino Acids. 2021 Feb 13;53(3):323–31. doi: 10.1007/s00726-021-02951-7 (PMC7979671; doi:10.1007/s00726-021-02951-7)
Supplement: Supplementary file 1 — Supplementary file1 (PDF 371 KB) [file 726_2021_2951_MOESM1_ESM.pdf]

Supplementary Material

Isolation of pedopeptin A and B.

Pedopeptin A and B[1] were isolated from cultures of two different bacterial isolates, UP1437 and UP1427, respectively. These strains were obtained in a larger study directed towards isolation of multidrug-resistant *Pedobacter* sp. from environmental samples, using combinations of multiple antibiotic drugs as selection approach (unpublished data). The strain UP1427 was isolated from a soil sample collected at a site (68°28'24.7"N 18°49'5.0"E) in Gävleborg County, Sweden, whereas UP1437 was from a site (57°19'47.9"N 18°42'43.0"E) in Norrbotten County, Sweden, north of the Arctic Circle. The samples were collected by pressing or digging a sterile centrifuge tube into the soil surface. Prior to isolation of bacterial strains the samples were stored cold (4˚ C) until used for isolation of bacterial strains.

A modified CN medium[2] was used for isolating bacteria. The modified medium denoted NBCA (Nutrient Broth/Casamino Acid agar medium) contained: 1 g Difco Nutrient Broth (BD Difco Ltd., Detroit, MI, USA), 1 g Casamino Acids (BD Difco Ltd.), 15 g Bacto agar (Saveen & Werner, Limhamn, Sweden) in 1 L of deionized water was after autoclaving and cooling the medium to approximately 50 °C supplemented with fungicides and various antibiotics. . Two fungicides, cycloheximide and nystatin, were, always, added to the final concentration of 100 mg/L and 10 mg/L, respectively in order to prevent growth of fungi and combinations of antibiotics were applied to allow isolation multidrug-resistant bacterial strains. UP1427 was isolated in the presence of nalidixic acid (20 mg/L), ampicillin (20 mg/L), and kanamycin (20 mg/L), and UP1437 in the presence of ciprofloxacin (1 mg/L), ampicillin (20 mg/L), and gentamicin (5 mg/L), respectively.

To isolate bacteria, approximately 4 g of each soil sample was mixed by vortexing with 40 mL of sterile phosphate-buffered saline (PBS). The mixture was allowed to settle for 10 minutes and then 1 mL of the supernatant was mixed with 9 mL of sterile PBS in a 15 mL sterile centrifuge tube. From this dilution, 0.1 mL was spread onto the NBCA isolation plates containing combinations of antibiotics. The plates were, then, incubated at 20°C for approximately one week in darkness to avoid degradation of light-sensitive antibiotics. Morphologically different bacterial colonies were afterwards re-streaked on Vegetable Peptone Broth Agar plates (VPA), containing 10 g Vegetable Peptone Broth (VPB, Oxoid Ltd, Basingstoke, Hampshire UK), and 15 g Bacto Agar (Saveen & Werner) in 1 L deionized water, purified, maintained at first on agar plates and then as deep-frozen cell stocks (-80 ˚C).

---

[1] Hirota-Takahata Y, Kozume S, Kuraya N, Fukuda D, Nakajima M, Ando O (2014) Pedopeptins, novel inhibitors of LPS: Taxonomy of producing organism, fermentation, isolation, physicochemical properties and structural elucidation. J Antib 67:243-251.

[2] Gavrish, E., Bollmann, A., Epstein, S., Lewis, K. (2008) A trap for in situ cultivation of filamentous actinobacteria. J. Microbiol. Methods, 72, 257-262.

UP1427 and UP1437 were identified as closest to *Pedobacter cryoconitis* A37 and *P. lusitanus* NL19, respectively, by means of sequencing of the 16S rRNA genes. Briefly, colony PCR amplification of the 16S rRNA gene was performed with the universal primers 27F (AGAGTTTGATCMTGGCTCAG) and 1492R (TACGGYTACCTTGTTACGACTT) followed by purification of amplified PCR-products (QIAquick PCR purifications kit, QIAGEN). Sequencing was then performed by Macrogen Europe (EZ-sequencing, Amsterdam, Netherlands). The resulting DNA sequencing chromatograms were assembled using the Geneious R8 software and manually inspected (Biomatters Ltd., Auckland, New Zealand). The resulting sequences were submitted to an online Nucleotide BLAST search (https://blast.ncbi.nlm.nih.gov/Blast.cgi) against 16S rRNA sequences (Bacteria and Archeae) to retrieve the closest possible identity of the isolate. The 16S rRNA sequences of both isolates were deposited at GenBank (UP1427 accession number MW332367 and UP1437 accession number MW332374).

For isolation of pedopeptin A and B, the strains UP1437 and UP1427were grown as liquid cultures (150 mL in 500-mL Erlenmeyer flasks) using half strength Vegetable Peptone Broth (VPB; 15 g VPB (Oxoid Ltd) in 1 L deionised water). Cultures were either started by transferring a loop (10 µL) of 24-48 h-old bacterial colonies grown on VPA plates or by transferring 100 µL of deep-frozen isolate stock per 150 mL VPB. The cultures were incubated on a rotary shaker (130 rpm) for 96-120 h at 20 °C in darkness. To collect extracellular metabolites, one sterile nylon mesh bag containing a polymeric resin, Sepabeads® SP850 (Sigma-Aldrich), was submerged in each actively growing culture16 to 24 hours after inoculation (approximately 8 g of resin per bag). At harvest, each adsorbent resin bag was washed with deionized water to remove bacterial cells and culture resins. Each bag was subsequently extracted with 2 × 20 mL MeOH and 2 × 20 mL MeCN, and the extracts were pooled and dried in a vacuum centrifuge. The dried extracts were dissolved in 1 mL 50% MeCN and following centrifugation (13 000 rpm for 5 min), 1-mL samples were fractionated on a reversed phase preparative HPLC column (Luna Omega PS C18, 21.2 × 100 mm, 5 µm), eluted with a gradient of MeCN in water (10-95% MeCN in 15 min and a hold at 95% MeCN for 10 min, at 10 mL/min) with 0.2% formic acid. The eluent was monitored at 210 nm and fractions (2 mL) were collected in deep-well plates (start after 5 min). Analysis by UHPLC-MS (as previously described[3]) showed fractions 26-28 from UP1437 to contain a compound with *m/z* 372.5532 (3+) in accord with pedopeptin A[1] (theory *m/z* 372.5535, 3+), whereas fractions 30-35 from UP1427 contained a compound with *m/z* 367.2222 (3+) in line with pedopeptin B[1] (theory *m/z* 367.2219, 3+). The fractions containing pedopeptin A and B, respectively, were pooled and dried in a vacuum centrifuge.

Pedopeptin A was further purified by preparative HPLC on a Hypercarb column (21.2 × 100 mm) using a gradient of MeCN in water (27.5-77.5% MeCN in 17 min and a 7-min hold at 77.5% MeCN, at 10 ml/min) with 0.2% formic acid. Fractions were collected as above, and fractions 22-23 were pooled and dried to give 0.36 mg pedopeptin A. Analysis by NMR in 10 mM DCl, gave data in accord with literature data for pedopeptin A.[1] The structure was also verified by MSMS

[3] Nord S, Bjerketorp J, Levenfors JJ, Cao S, Strömstedt A, Guss B, Larsson R, Hughes D, Öberg B, Broberg A (2020) Isopedopeptins A-H: Cationic Cyclic Lipodepsipeptides from *Pedobacter cryoconitis* UP508 targeting WHO top-priority carbapenem-resistant bacteria. ACS Chem Biol 15:2937-2944.

(Supplementary Figure 1) after treatment with 0.2% NaOMe in MeOH for 30 min (as previously described[3]). Pedopeptin B was further purified by preparative HPLC (Luna Omega PS C18, 21.2 × 100 mm, 5 μm), eluted with a gradient of MeCN in water (20-50% MeCN in 17 min and a hold at 50% MeCN for 5 min, at 10 mL/min) with 0.2% formic acid. Fractions 66-70 were pooled and dried to give 0.40 mg pedopeptin B. The structure of pedopeptin B was determined by MSMS after treatment with 0.2% NaOMe in MeOH for 30 min (Supplementary Figure 1). The configuration of the amino acids in pedopeptin A and B were found to be as previously described[1] by the advanced Marfey's method.[4,5]

---

[4] Harada K, Fujii K, Mayumi T, Hibino Y, Suzuki M, Ikai Y, Oka H (1995) Constituent Amino Acids in Peptide --- Advanced Marfey's Method. Tetrahedron Lett 36:1515-1518.

[5] Fujii K, Ikai Y, Mayumi T, Oka H, Suzuki M, Harada K (1997) A Nonempirical Method Using LC/MS for Determination of the Absolute Configuration of Constituent Amino Acids in a Peptide: Elucidation of Limitations of Marfey's Method and of Its Separation Mechanism. Anal Chem 69:3346-3352.

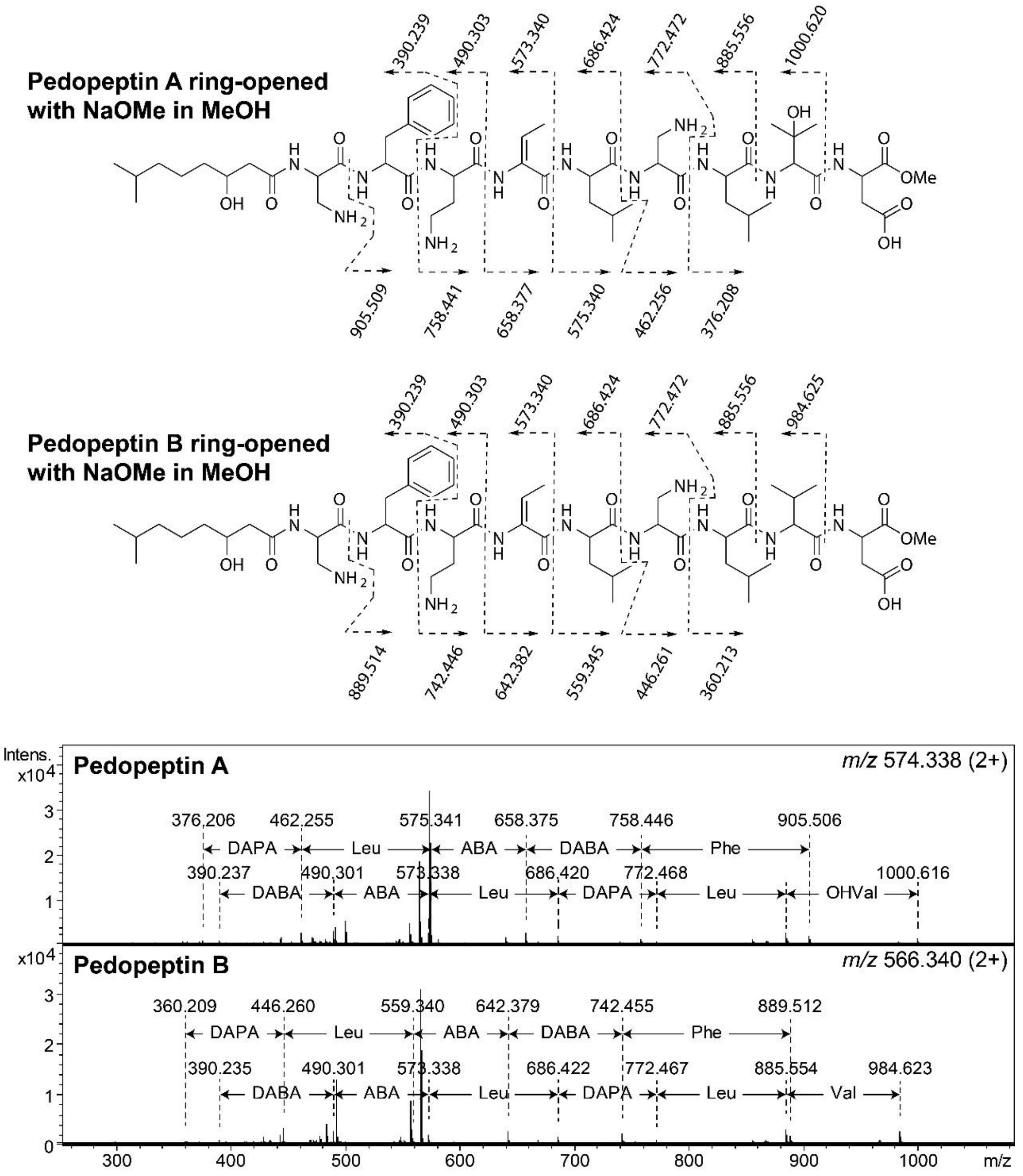

Supplementary Figure 1. Top: Structures of pedopeptin A and B after ring-opening with NaOMe in MeOH, with theoretical *m/z* values for selected Y- and B-series ions. **Bottom:** MSMS spectra from analysis of pedopeptin A and B after ring-opening with NaOMe in MeOH. In each spectrum, B-ions with corresponding amino acid sequence are shown on bottom, and Y-ions with corresponding amino acid sequence are shown on top. Precursor ions are shown in the top right corner in each spectrum.
